# Warm water immersion in patients with chronic heart failure: a pilot study: Shah immerse: HF

**DOI:** 10.1007/s00392-018-1376-2

**Published:** 2018-09-28

**Authors:** Parin Shah, Pierpaolo Pellicori, Anna Kallvikbacka-Bennett, Jufen Zhang, Daniel Pan, Andrew L. Clark

**Affiliations:** 1 Department of Cardiology, Hull York Medical School, Hull and East Yorkshire Medical Research and Teaching Centre, Castle Hill Hospital, Cottingham, Kingston upon Hull, HU16 5JQ UK; 2 Clinical Trials Unit, Postgraduate Medical Institute, Faculty of Medical Science, Anglia Ruskin University, Bishop Hall Lane, Chelmsford, Essex CM1 1SQ UK

**Keywords:** Heart failure, Exercise, Water immersion, Swimming

## Abstract

**Background:**

Patients with chronic conditions, such as heart failure, swim regularly and most rehabilitation exercises are conducted in warm hydrotherapy pools. However, little is known about the acute effects of warm water immersion (WWI) on cardiac haemodynamics in patients with chronic heart failure (CHF).

**Methods:**

Seventeen patients with CHF (NYHA I and II; mean age 67 years, 88% male, mean left ventricular ejection fraction 33%) and 10 age-matched normal subjects were immersed up to the neck in a hydrotherapy pool (33–35 °C). Cardiac haemodynamics were measured non-invasively, and echocardiography was performed at baseline, during WWI, 3 min after kicking in the supine position and after emerging.

**Results:**

In patients with CHF, compared to baseline, WWI immediately increased stroke volume (SV, mean ± standard deviation; from 65 ± 21 to 82 ± 22 mL, *p* < 0.001), cardiac output (CO, from 4.4 ± 1.4 to 5.7 ± 1.6 L/min, *p* < 0.001) and cardiac index (CI, from 2.3 ± 0.6 to 2.9 ± 0.70 L/min/m², *p* < 0.001) with decreased systemic vascular resistance (from 1881 ± 582 to 1258 ± 332 dynes/s/cm^5^, *p* < 0.001) and systolic blood pressure (132 ± 21 to 115 ± 23 mmHg, *p* < 0.001). The haemodynamic changes persisted for 15 min of WWI. In normal subjects, compared to baseline, WWI increased SV (from 68 ± 11 to 80 ± 18 mL, *p* < 0.001), CO (from 5.1 ± 1.9 to 5.7 ± 1.8 L/min, *p* < 0.001) and CI (from 2.7 ± 0.9 to 2.9 ± 1.0 L/min/m², *p* < 0.001).In patients with CHF, compared to baseline, WWI caused an increase in left atrial volume (from 57 ± 44 to 72 ± 46 mL, *p* = 0.04), without any changes in left ventricular size or function or amino terminal pro B-type natriuretic peptide.

**Conclusions:**

In patients with CHF, WWI causes an acute increase in cardiac output and a fall in systemic vascular resistance.

**Clinical trial registration:**

ClinicalTrials.gov (Identifier: NCT02949544) https://clinicaltrials.gov/ct2/show/NCT02949544?cond=NCT02949544&rank=1.

## Introduction

Exercise training is recommended for patients with chronic heart failure (CHF) [1–4], not only to improve exercise capacity but also for its beneficial effects on morbidity and mortality [5–7]. Although symptom-limiting exercise is safe in patients with heart failure [8], factors such as advanced age [9, 10], and comorbidities such as osteoarthritis, hinder exercise training on a treadmill or a cycle in patients with CHF. Swimming is common in the United Kingdom and has the advantage of providing buoyancy to reduce impact on joints [11]. Swimming in patients with arthritis improves mobility, strength and cardiovascular fitness without any joint discomfort [12].

Whether swimming is safe in patients with CHF has not been answered comprehensively in previous studies of water immersion or swimming [13]. When normal subjects are immersed up to the neck in water, the hydrostatic pressure forces approximately 700 mL of blood pooled in the periphery to the cardiothoracic space [14]. The increased preload to the heart increases stroke volume by 34–50% in normal subjects [14, 15]. However, this increased preload to the failing heart may precipitate pulmonary oedema. Currently, only the Scottish guidelines on the management of CHF mention swimming to warn against it in patients with New York Heart Association (NYHA) class III and IV symptoms [4].

The aim of the present study was to assess the acute hemodynamic, echocardiographic and amino terminal pro B-type natriuretic peptide (NTproBNP) changes during warm water immersion (WWI) in patients with CHF.

## Methods

Ambulatory patients with an established diagnosis of CHF, on stable treatment for more than 3 months were enrolled from a community heart failure clinic. Patients with severe symptoms (NYHA class IV), weight over 120 kilograms, hospitalised within last 6 weeks or with a contraindication to WWI (epilepsy, recent hypoglycaemia, intravenous line or urinary catheter) were excluded from the study. Controls were normal subjects, over 60 years of age, who were already consented to take part as healthy volunteers in a local observational research program. Normal subjects had to have a left ventricular ejection fraction (LVEF) ≥ 50% on echocardiography.

The research conforms to the Helsinki declaration and ethics approval was granted by an external research ethics committee. The trial was registered on the ClinicalTrials.gov website (Identifier: NCT02949544) and all participants gave their written informed consent.

### Water immersion protocol

**Fig. 1:**
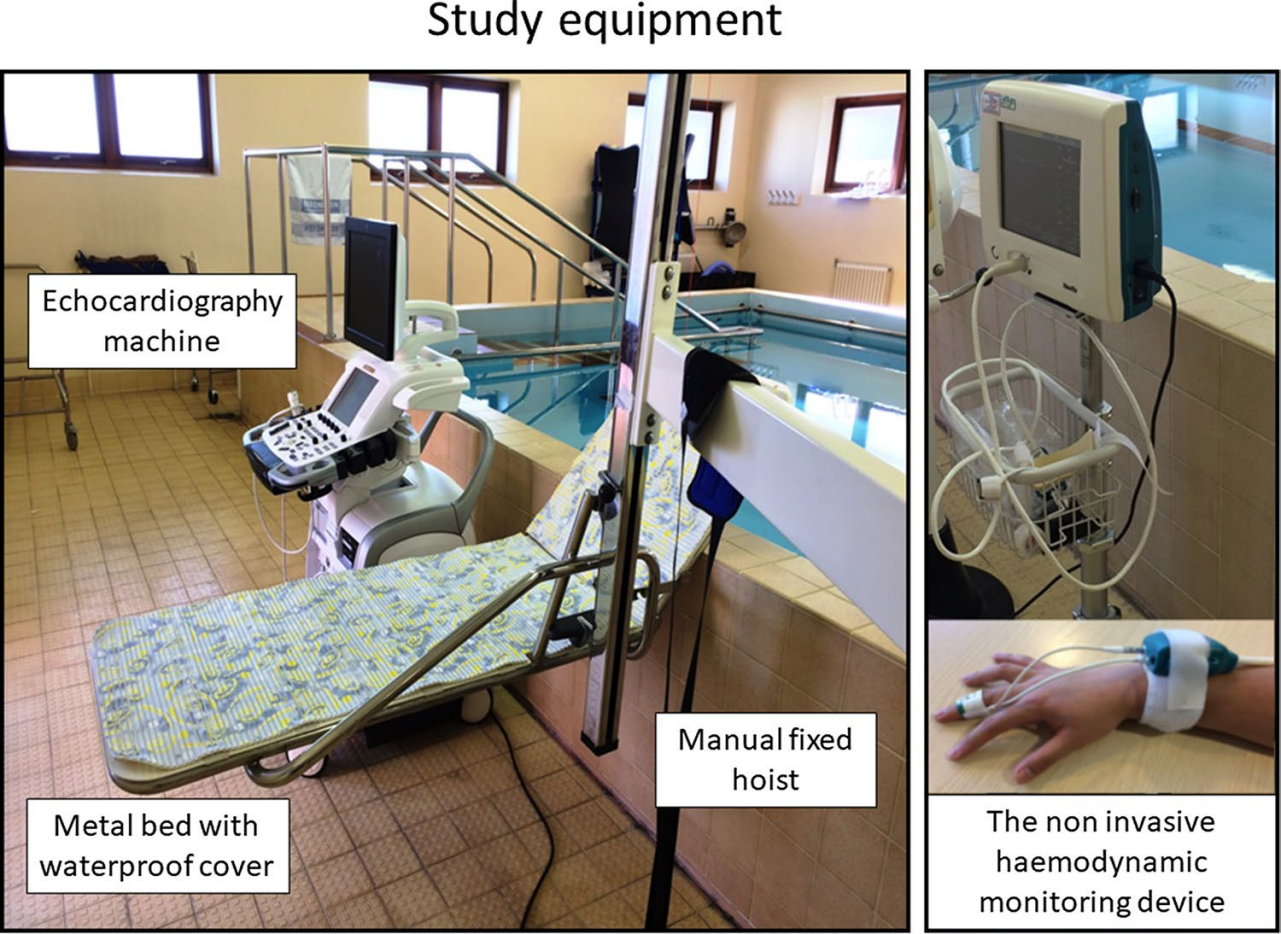
Study equipment. Echocardiography machine: GE Vivid E9, Hatfield, Hertfordshire, UK. Non-invasive haemodynamic monitoring device (Nexfin, BMeye, Amsterdam, The Netherlands) with an inflatable finger cuff was attached to the mid finger of participant’s left hand. Hydrotherapy pool (maintained at a temperature between 33 and 35 °C)

The study was conducted in a hydrotherapy pool on the hospital site. The pool temperature was maintained between 33 and 35 °C and the outside temperature was maintained at 21 °C. A metal bed designed to be immersed in water was attached to a manual hoist fixed next to the pool. Participants remained in supine position on the bed throughout the study and the patients were immersed and removed from the water using the hoist. (Fig. 1) Supine position was chosen as this was the best position to mimic swimming posture and also enable non-invasive haemodynamic measurements and echocardiography whilst immersed.

Baseline measurements, including a blood sample for NTproBNP analysis, and echocardiography (GE Vivid E9, Hatfield, Hertfordshire, UK) were conducted after participants were comfortable on the bed for 10–15 min. A non-invasive haemodynamic monitoring device (Nexfin, BMeye, Amsterdam, The Netherlands) was used to continuously monitor cardiac haemodynamics. This device has been validated against invasive methods of monitoring cardiac haemodynamics in studies of patients with heart failure and critically ill patients [16–18]. An inflatable finger cuff was attached to the mid finger of participant’s left hand (Fig. 1) and during immersion the left hand was rested on a floater to ensure the device remained out of the water. The device continuously recorded cardiac haemodynamics from baseline to the end of the study. The reference level for the non-invasive haemodynamic device was at the surface of the water throughout, and thus very slightly higher than the level of the heart. The measurements outside the pool were made with the reference level in the same relative position before immersion. A non-invasive device (VENUS 2000 CVP monitor, Mespere LifeScience Inc, Waterloo, Canada) was used to measure central venous pressure (CVP) using a small adhesive neck sensor placed over the external jugular vein. The device uses near infrared spectroscopy to determine the pressure in the external jugular vein and its clinical use has been validated in critically ill patients with cardiovascular conditions, and in out-patients setting for patient with heart failure [19–21].

The participants were then gradually immersed up to their neck into the hydrotherapy pool in supine position. Echocardiography was repeated at 1 and 15 min following WWI with the ultrasound probe covered with a light polythene bag to ensure it remained water proof. After 15 min of WWI, the participants were asked to perform gentle exercise (3 min of kicking at an speed of 40 repetitions per minute) whilst remaining in supine position on the bed. The exercises performed was hip extension and flexion with a straight leg. At the end of 3 min of exercise, echocardiography was repeated. Once all the echocardiographic and haemodynamic readings were recorded the participants were lifted out of the water. Haemodynamic readings, symptom scores, blood sample for NTproBNP and echocardiography were repeated whilst participants remained in supine position on the bed 3 min after emerging from the hydrotherapy pool.

All the electric devices used were checked by University engineers to ensure they were safe for use near water. Additional circuit breaking sockets were installed in case of any power surges or short circuits.

From the non-invasive haemodynamic and central venous pressure monitoring devices, the following variables were recorded at baseline, 1 min WWI, 15 min WWI, 3 min after exercise and after 3 min recovery: heart rate (HR), blood pressure (BP), stroke volume (SV), cardiac output (CO), cardiac index (CI), systemic vascular resistance (SVR) and central venous pressure. An average of five measured values for each haemodynamic variable was taken. All echocardiographic exams were performed by the lead cardiac sonographer in the Department of Academic Cardiology (AB), subsequently stored on DVDs, and finally reviewed off-line by a single experienced operator blind to the various phases of the study (PS).The following echocardiographic variables were measured: left ventricular end diastolic (EDV) and end systolic volumes (ESV), left ventricular ejection fraction (LVEF), left atrial diameter (LAD) and volume (LAV) tricuspid annular plane systolic excursion (TAPSE), systolic tricuspid regurgitation (TR) pressure gradient, inferior vena cava (IVC) diameter. Patients’ modified Borg score, Canadian cardiovascular society angina grading scale, fatigue symptom inventory symptom scores were taken at baseline and recovery.

### Statistical analysis

Categorical data are presented as numbers and percentages; normally distributed continuous data as mean ± standard deviation (SD) and non-normally distributed continuous variables as median and interquartile range. The repeated measures ANOVA was used to assess the overall difference between related means of each variable and the Bonferroni correction was used for multiple testing errors. Log transformation of NTproBNP was used, given its not normal distribution. Primary and secondary endpoints are shown in graphs. All analyses were performed on SPSS (V.23.0), and statistical significance was assumed at *p* < 0.05 (two tailed).

## Results

### Baseline characteristics of patients with CHF and normal subjects

Baseline characteristics of patients with CHF and normal subjects are shown in Table 1. All but one of the patients had NYHA class II symptoms. Normal subjects were of a similar age, 90% were male and baseline cardiac haemodynamics were similar to patients with CHF.

**Table 1:** Baseline characteristics of patients with CHF and normal subjects

Baseline characteristic of participants			
Variables	Patients with CHF (*N* = 17)	Normal subjects (*N* = 10)	*p* value
Demographics			
Age	67 (12)	70 (10)	0.39
Male (%)	15 (88)	9 (90)	0.89
Weight (kg)	78 (13)	73 (10)	0.37
BMI (kg/m^2^)	26 (3)	24 (3)	0.19
SBP (mmHg)	129 (16)	143 (30)	0.14
NYHA class			
I (%)	1 (6)	10 (100)	< 0.001
II (%)	16 (94)	0 (0)	
Medical history			
Hypertension (%)	4 (24)	2 (20)	0.84
IHD (%)	12 (71)	0 (0)	< 0.01
Diabetes (%)	2 (12)	3 (30)	0.26
COPD (%)	2 (12)	0 (0)	0.28
Electrocardiogram			
Sinus	14 (82)	10 (100)	0.17
HR (beats/min)	65 (10)	66 (12)	0.87
Echocardiography			
LVEDV (mL)	189 (64)	88 (37)	< 0.001
LVEF (%)	33 (9)	58 (8)	< 0.001
LAD (mm)	4 (0.9)	3.5 (0.6)	0.15
LAV (mL)	57 (44)	36 (22)	0.2
TAPSE(mm)	1.9 (0.6)	2.7 (0.5)	0
Peak TR gradient (mmHg)	18 (9)	15 (8)	0.49
IVC (cm)	1.6 (0.5)	1.4 (0.4)	0.31
Blood results			
Hb (g/L)	13.4 (1.3)	14.2 (1.2)	0.09
Creatinine (µmol/L)	114 (24)	73 (13)	< 0.001
NTproBNP (ng/L)	558 (323–1140)	82 (42–119)	< 0.01
Medications and devices			
ACEi/ ARB (%)	15 (88)	1 (10)	< 0.001
Beta blocker (%)	15 (88)	0 (0)	< 0.001
MRA (%)	13 (77)	0 (0)	< 0.001
Loop diuretic (%)	14 (82)	0 (0)	< 0.001
CRT (%)	7 (41)	0 (0)	0.02

*CHF* chronic heart failure, *BMI* body mass index, *SBP* systolic blood pressure, *NYHA* New York Heart Association, *IHD* ischaemic heart disease, *COPD* chronic obstructive pulmonary disease, *HR* heart rate, *LVEDV* left ventricular end diastolic volume, *LVEF* left ventricular ejection fraction, *LAD* left atrial diameter, *LAV* left atrial volume, *TAPSE* tricuspid annular plane systolic excursion, *TR* tricuspid regurgitation, *IVC* inferior vena cava, *Hb* haemoglobin, *NTproBNP* amino terminal pro brain type natriuretic peptide, *ACEi* angiotensin converting enzyme inhibitor, *ARB* angiotensin receptor blocker, *MRA* mineralocorticoid receptor antagonist, *CRT* cardiac resynchronization therapy

### Haemodynamic changes with warm water immersion and exercise

**Fig. 2:**
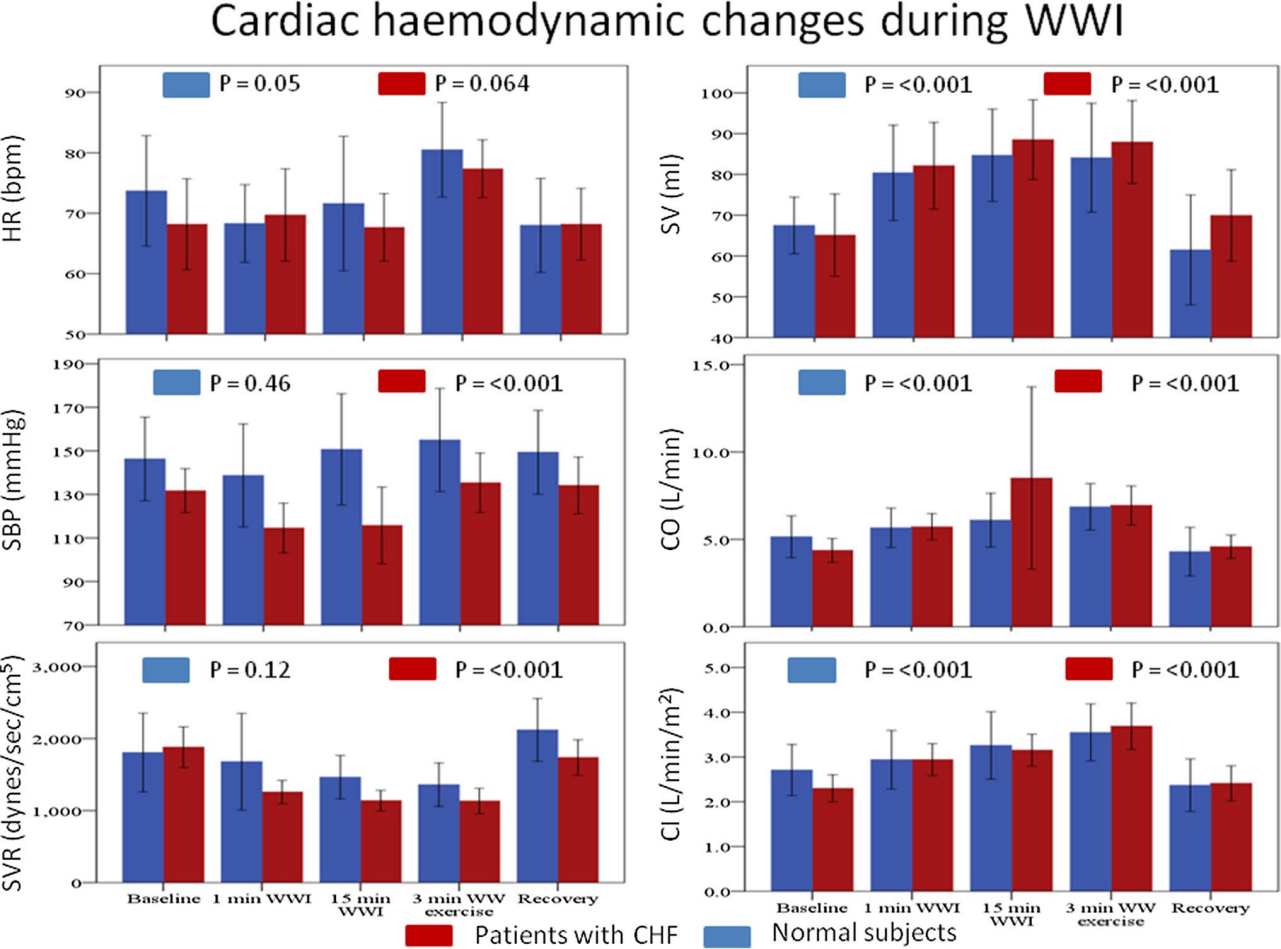
Cardiac haemodynamic changes during WWI measured by non-invasive haemodynamic monitoring device (Nexfin, BMeye, Amsterdam, The Netherlands). Cardiac haemodynamic changes were measured at baseline, 1 min WWI, 15 min WWI, 3 min after WW exercise, at recovery. *HR* heart rate, *SBP* systolic blood pressure, *SVR* systemic vascular resistance, *SV* stroke volume, *CO* cardiac output, *CI* cardiac index, *WWI* warm water immersion, *WW* warm water, *CHF* chronic heart failure

**Fig. 3:**
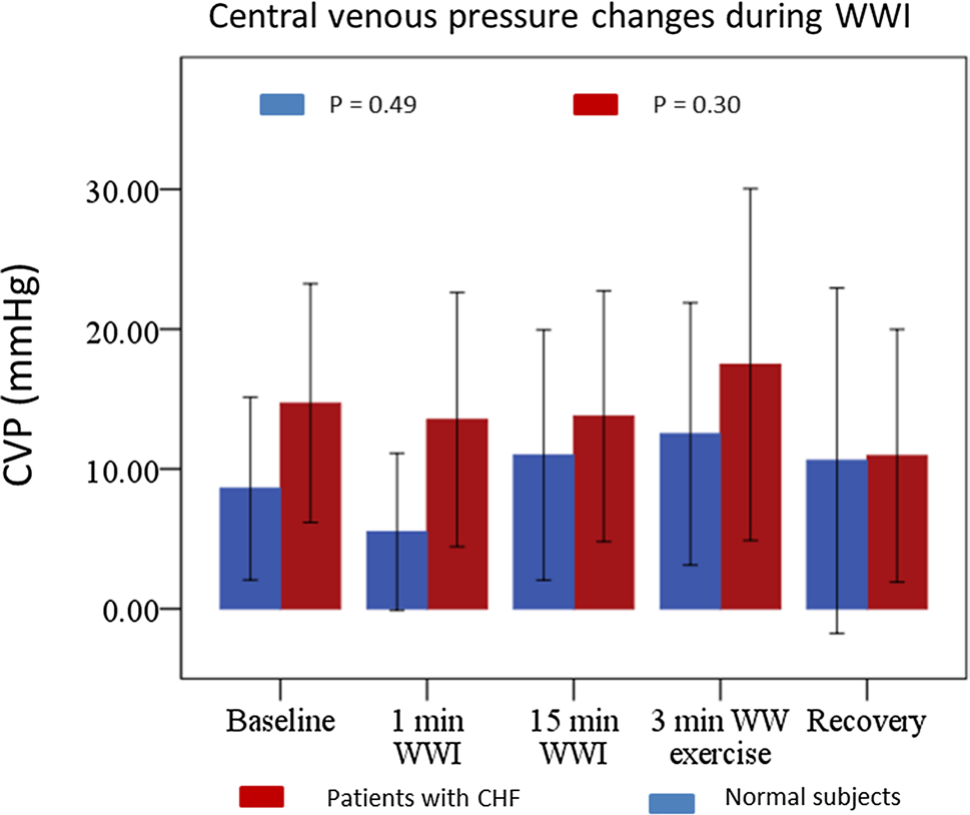
CVP changes during WWI. CVP changes were measured at baseline, 1 min WWI, 15 min WWI, 3 min after WW exercise, at recovery. *CVP* central venous pressure, *WWI* warm water immersion, *WW* warm water, *CHF* chronic heart failure

In patients with CHF, and compared to baseline measurements, WWI caused a significant, immediate (1 min) and sustained (after 15 min) increase in SV, CO, CI and decrease in BP and SVR, with no change in HR or CVP. The 3 min of kicking led to a further increase in HR, BP, CI and CO, with no change in SV, SVR or CVP (Figs. 2, 3).

In normal subjects, WWI caused a significant, immediate (1 min) and sustained (15 min) increase in SV, CO and CI with no change in HR, BP, SVR or CVP. The 3 min of kicking led to an increase in HR, CO and CVP with no change in BP, SV, CI or SVR (Figs. 2, 3).

### Echocardiographic changes with warm water immersion and exercise

**Fig. 4:**
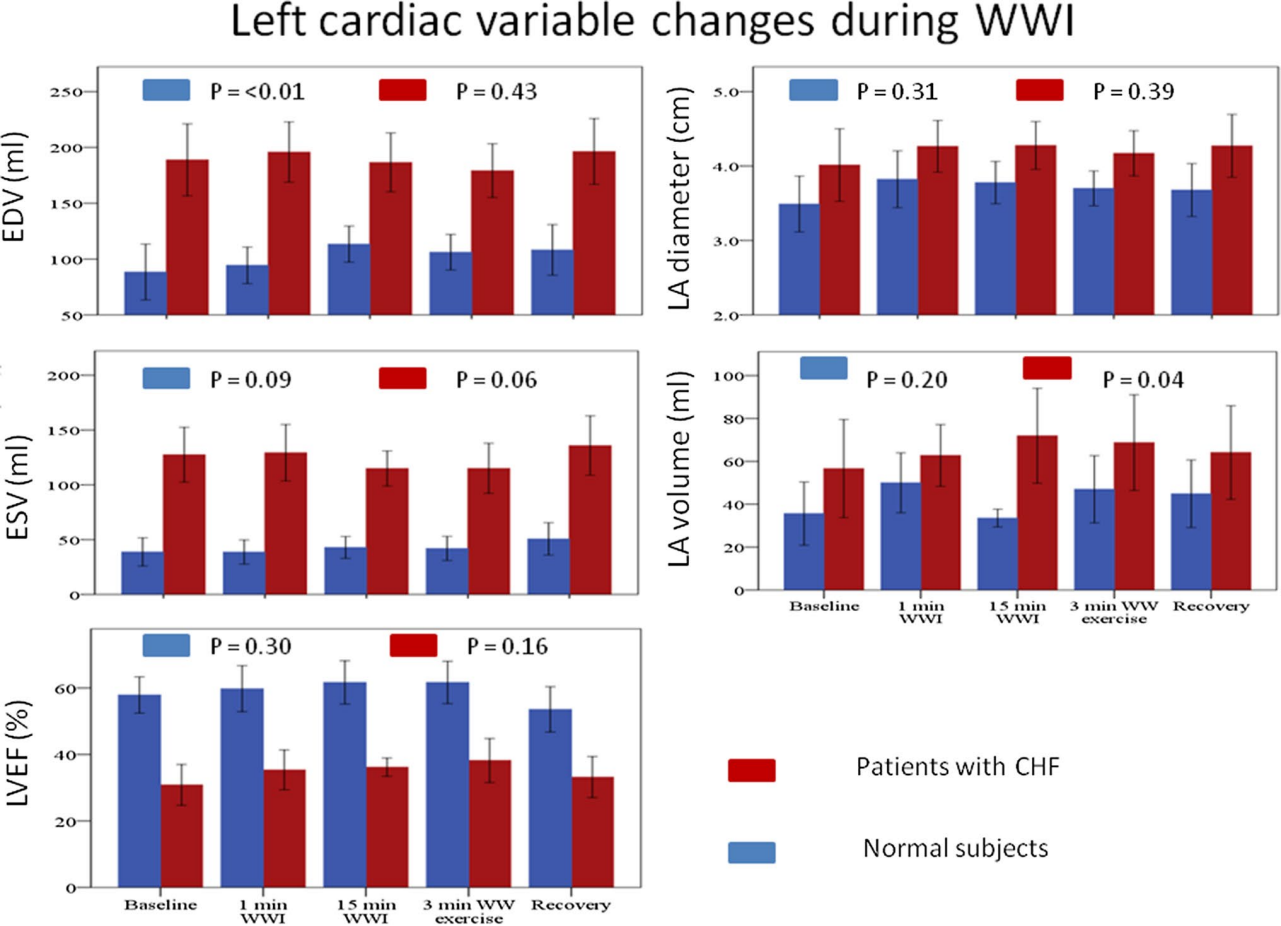
Left cardiac variable changes during WWI. Left cardiac variables changes were measured at baseline, 1 min WWI, 15 min WWI, 3 min after WW exercise, at recovery. *EDV* End diastolic volume, *ESV* end systolic volume, *LVEF* left ventricular ejection fraction, *LA* left atrial, *WWI* warm water immersion, *WW* warm water, *CHF* chronic heart failure

**Fig. 5:**
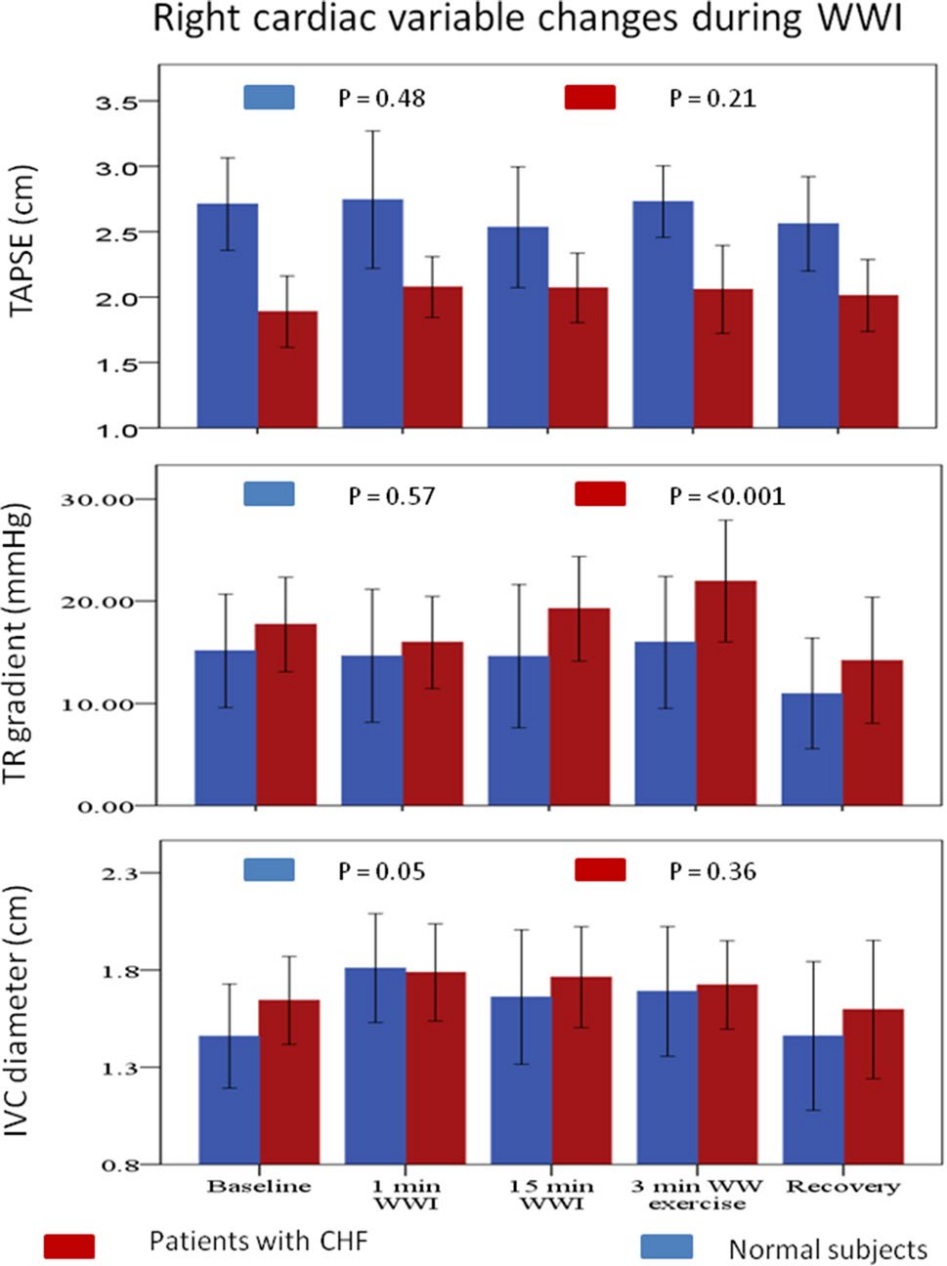
Right cardiac variable changes during WWI. Right cardiac variables changes were measured at baseline, 1 min WWI, 15 min WWI, 3 min after WW exercise, at recovery. TAPSE: tricuspid annular plane systolic excursion, *TR* tricuspid regurgitation, *IVC* inferior vena cava, *WWI* warm water immersion, *WW* warm water, *CHF* chronic heart failure

In patients with CHF, WWI for 15 min led to a significant increase in LAV. The 3 min of kicking led to an increase in estimated pulmonary artery systolic pressure (Figs. 4, 5).

In normal subjects, WWI for 15 min significantly increased left ventricular EDV. There were no significant changes in echocardiographic variables after 3 min of kicking (Figs. 4, 5).

### NTproBNP and symptom changes with warm water immersion and exercise

There was no significant difference in NTproBNP levels between baseline and recovery in patients with CHF [558 (IQR: 323–1140) to 642 (IQR: 309–1187) ng/L, *p* = 0.21] or normal subjects [82 (42–119) to 84 (43–118) ng/L, *p* = 0.08]. None of the subjects experienced any adverse events during water immersion. None of the patients reported any change in any symptom (shortness of breath, chest pain or fatigue) during immersion or recovery.

## Discussion

We found that WWI in patients with CHF increases cardiac output. The mechanism for the haemodynamic changes is presumably that hydrostatic pressure increased cardiac preload and atrial volumes. WWI caused systemic vasodilation, leading to a fall in blood pressure which, in turn, causes a decreased left ventricular afterload.

Our results are similar to those reported by other researchers. In a study of nine patients with CHF, WWI (34 °C) up to the xiphoid process increased central venous pressure, and as in our study, increased left atrial diameter (by echocardiography), CI and SV index with a decrease in SVR [22]. In a study of 13 patients with CHF (mean LVEF 32%) and more severe symptoms (77% in NYHA class 3), WWI (33–34 °C) up to the sternal notch increased LVEF, SV and CO but also worsened left ventricular diastolic function (increasing trans mitral Doppler *E*/*A* ratio), with no effect on blood pressure [23]. 5 min of seated reciprocal unilateral knee-extensions in water increased CO, SBP and HR, and was tolerated by patients [23]. Similarly, in a study of 18 patients with CHF (LVEF 31%, 50% in NYHA class 3), WWI (34 °C) significantly increased SV and CO but decreased HR, BP and SVR. WWI also led to an increase in left ventricular end diastolic and systolic volumes, estimated pulmonary capillary wedge pressure and left ventricular ejection fraction [24].

All the previous studies of WWI on cardiac haemodynamic had patients in sitting or standing positions during WWI. We positioned the participants in supine position because this better replicates the posture during swimming [22–24].

Warmer or colder water temperatures might lead to more pronounced, or different effects, on cardiac haemodynamics and heart function. Hot water immersion (41 °C) for 10 min significantly decreased left ventricular and atrial size, increased LVEF and decreased mitral regurgitation in a study of 34 patients with CHF and severe symptoms (LVEF 25%, 94% in NYHA class III/IV). The changes persisted for 30 min after emerging from water [25]. Mean pulmonary artery pressure, mean pulmonary capillary wedge pressure, and mean right atrial pressure rose substantially during hot water immersion but did not cause any symptoms. In two studies, cold-water immersion (12–22°C) for a few seconds increased cardiac output and blood pressure but change in heart rate was variable [26, 27].

Swimming is a popular form of exercise in the United Kingdom [11]. Whether swimming is safe in patients with heart failure is still not known [13, 14]. Swimming-induced pulmonary oedema (SIPE) is a rare occurrence seen in athletes undertaking strenuous exercise [28, 29]. In all studies of water immersion in patients with CHF there have been no reports of SIPE, although the time of water immersion and exercise has been limited to a few minutes in a controlled environment.

In previous studies, warm water immersion up to the neck in standing position for 5–10 min, has increased central venous pressure [30, 31]. We found that central venous pressure did not increase and pulmonary artery systolic pressure increases only after 3 min of gentle exercise in patients with CHF (without precipitating any symptoms), but not in normal subjects. The pulmonary artery systolic pressure rapidly returned to baseline after WWI. Reassuringly, the haemodynamic changes did not lead to an increase in NTproBNP plasma levels. In studies looking at the effect of WWI on natriuretic peptides in normal subjects, atrial natriuretic peptide significantly increased without any change in brain natriuretic peptide [32, 33]. There have been no studies looking at the acute change in natriuretic peptides during water immersion in patients with heart failure. Whether prolonged immersion or swimming causes a more sustained increase in systolic pulmonary pressure and symptoms in patients with CHF requires further studies.

### Limitations

Our study was conducted in a controlled, indoor thermoneutral hydrotherapy pool, and therefore, the results cannot be translated to swimming in different environmental conditions. The studied population was small, and patients had only mild symptoms; thus, results of this study cannot be generalised to all patients with CHF, particularly to those with more severe disease. Although majority of the patients swim in prone, we were limited to investigate patients in supine position to enable echocardiography. The workload was not standardized and not adjusted for maximal exercise capacity. It was chosen to allow all participants to be able to conduct 3 min of exercise with continuous haemodynamic monitoring. We did not measure NTproBNP levels after a few hours from completing the study, or troponin levels, which might have provided further information about any delayed effect of WWI with exercise on the myocardium.

## Conclusion

In patients with CHF, WWI causes an acute increase in cardiac output and a fall in vascular resistance. The changes were well-tolerated. Whether swimming can be recommended as alternative to other forms of exercise or rehabilitation in patients with CHF needs to be studied further.
